# Extensive Epigenetic Changes Accompany Terminal Differentiation of Mouse Hepatocytes After Birth

**DOI:** 10.1534/g3.116.034785

**Published:** 2016-09-21

**Authors:** Matthew V. Cannon, Genay Pilarowski, Xiuli Liu, David Serre

**Affiliations:** * Genomic Medicine Institute, Lerner Research Institute, Cleveland Clinic, Ohio 44195; † Department of Anatomic Pathology, Cleveland Clinic, Ohio 44195

**Keywords:** DNA methylation, epigenetics, liver development, differentiation, hepatocytes

## Abstract

DNA methylation is traditionally thought to be established during early development and to remain mostly unchanged thereafter in healthy tissues, although recent studies have shown that this epigenetic mark can be more dynamic. Epigenetic changes occur in the liver after birth, but the timing and underlying biological processes leading to DNA methylation changes are not well understood. We hypothesized that this epigenetic reprogramming was the result of terminal differentiation of hepatocyte precursors. Using genomic approaches, we characterized the DNA methylation patterns in mouse liver from E18.5 until adulthood to determine if the timing of the DNA methylation change overlaps with hepatocyte terminal differentiation, and to examine the genomic context of these changes and identify the regulatory elements involved. Out of 271,325 CpGs analyzed throughout the genome, 214,709 CpGs changed DNA methylation by more than 5% (*e.g.*, from 5 to 10% methylation) between E18.5 and 9 wk of age, and 18,863 CpGs changed DNA methylation by more than 30%. Genome-scale data from six time points between E18.5 and P20 show that DNA methylation changes coincided with the terminal differentiation of hepatoblasts into hepatocytes. We also showed that epigenetic reprogramming occurred primarily in intergenic enhancer regions while gene promoters were less affected. Our data suggest that normal postnatal hepatic development and maturation involves extensive epigenetic remodeling of the genome, and that enhancers play a key role in controlling the transition from hepatoblasts to fully differentiated hepatocytes. Our study provides a solid foundation to support future research aimed at further revealing the role of epigenetics in stem cell biology.

Many cellular processes, including normal growth and development, involve carefully regulated changes in gene expression. Epigenetic modifications, particularly histone modifications and DNA methylation, are known to play a critical role in regulating gene expression. While histone modifications are dynamically regulated, DNA methylation of CpG dinucleotides is considered to be mostly static in adult cells after establishment of these marks during early development (Smith and Meissner 2013). The methylation patterns in developing cells are erased before implantation of the blastocyst (with the exception of imprinted regions), and the reestablishment of DNA methylation afterward is thought to set up the epigenetic programs of each cell lineage (Smith and Meissner 2013). Epigenetic changes during later cellular differentiation have been documented but these mostly occur before birth (Smith and Meissner 2013; Perera and Herbstman 2011; Reik *et al.* 2001; Smith *et al.* 2012; Wang *et al.* 2012; Stadler *et al.* 2011; Meissner *et al.* 2008; Bock *et al.* 2012; Gifford *et al.* 2013; Xie *et al.* 2013) (*e.g.*, the differentiation of embryonic stem cells). However, recent studies have revealed that DNA methylation is more fluid during postnatal development than previously thought (Bock *et al.* 2012; Reizel *et al.* 2015). For example, several studies have shown postnatal changes in DNA methylation in mouse liver (Waterland *et al.* 2009; Reizel *et al.* 2015).

There are, however, many unresolved questions relating to postnatal DNA methylation changes in the liver, including the mechanism(s) underlying these epigenetic changes, the timing of these changes, and the extent and precise genomic context where these changes occur. We hypothesized that terminal differentiation of hepatocytes was responsible for postnatal DNA methylation changes. Unfortunately, testing this hypothesis is complicated by (i) the lack of a robust *in vitro* model for hepatocyte differentiation and (ii) the complex cellular changes occurring *in vivo*, including efflux of hematopoietic stem cells (HSC), extensive cell division, and cell differentiation (Baron *et al.* 2013; Gordillo *et al.* 2015).

Here, we use Reduced Representation Bisulfite Sequencing (RRBS) to characterize DNA methylation changes throughout the genome of mouse liver samples collected immediately before birth and throughout the first weeks of life. Our study reveals that the timing of the DNA methylation coincides with hepatocyte terminal differentiation and occurs after HSC migration, and that the DNA methylation changes preferentially occur at specific genomic regulatory elements.

## Materials and Methods

### Tissue collection

We obtained C57BL/6J mice from Jackson Laboratories (Bar Harbor, Maine) at 3 wk of age and mated females at 8 wk of age. We housed mice in ventilated microisolator cages with a 14:10 light:dark cycle. We collected tissue samples from offspring at embryonic day 18.5 (E18.5), postnatal days P1, P5, P10, P15, and P20, and 9 wk. All tissues were stored in RNA later at −80°. We fed mice used for the initial RRBS experiments chow obtained from Research Diets Inc. (New Brunswick, NJ; D12328), which contains 11% fat from coconut oil (Cannon *et al.* 2014). Mice used for qRT-PCR and histology were fed standard chow. For our time-course study, we obtained tissues from E18.5, P1, P5, P10, P15, and P20 C57BL/6J mice directly from Jackson laboratories.

### Histological analysis

We prepared liver tissue excised from animals at each time point for histological analysis by embedding the tissue in Optimal Cutting Temperature compound (OCT). We immersed the sample in 2-methylbutane cooled by liquid nitrogen to freeze the samples. Frozen samples were stored at −80° until sectioning. We cut sections to 5 μm thickness and stained with Hematoxylin and Eosin (H&E) according to standard protocols. The percent of HSCs and hepatocytes for each slide was estimated by a trained pathologist by microscopy.

### DNA/RNA isolation

We isolated DNA and RNA from tissue samples using the Qiagen All-prep DNA/RNA kit (Venlo, Limburg) according to the manufacturer’s instructions.

### qRT-PCR

We used qRT-PCR to quantify gene expression of *Alb*, *Afp*, *Tdo2*, *Sds*, *Ccne1*, *Ccne2*, *Ccna2*, *Ccnb1*, and *Actb* (as a housekeeping gene). We treated RNA with DNase and performed reverse transcription with Invitrogen Superscript III reverse transcriptase (Carlsbad, CA). We conducted qRT-PCR on an Eppendorf Mastercycler RealPlex2 (Hamburg, Germany) machine using Qiagen QuantiTect SYBR green PCR reagents (Venlo, Limburg). Primer sequences are provided in Supplemental Material, Table S1. qRT-PCR data were normalized to *Actb* and either the E18.5 or 9 wk time point was used as a reference (whichever was lower). Differences between time points were tested using Student’s *t*-tests with a Bonferroni correction for multiple comparisons.

### RRBS library preparations

To measure DNA methylation at numerous loci across the genome, we performed RRBS experiments (adapted from Gu *et al.* 2011) separately on two mouse cohorts. This protocol allows reproducible, base pair resolution quantification of DNA methylation at a large subset of CpGs across the genome. Briefly, we digested genomic DNA extracted from each mouse liver with the *Msp*I restriction enzyme, which cuts at the CCGG motif. We then prepared Illumina Truseq DNA libraries (San Diego, CA) according to the manufacturer’s instructions, with the exception that after adapter ligation, we pooled 12–18 samples with different barcodes and isolated fragments of ∼150–600 bp by gel extraction. We then bisulfite treated each pool using an Invitrogen MethylCode Bisulfite Conversion Kit (Carlsbad, CA). Finally, we performed enrichment PCR using the Truseq primers for 20 cycles. Each pool was sequenced on 2–3 lanes of a HiSequation 2500 instrument, producing 50 bp reads. In our discovery RRBS experiment, we analyzed liver samples from two time points [E18.5 (*n* = 5) and 9 wk old male mice (*n* = 10)]. A second independent experiment, the time-course dataset, was performed at a later time and included another mouse cohort and 90 samples from liver, heart, and muscle tissues collected at time points (5 per age per tissue) (E18.5, P1, P5, P10, P15, and P20).

### Locus-specific bisulfite sequencing (LSBS) library preparation

To validate the RRBS data, we selected 21 CpGs from 11 regions analyzed by RRBS and designed primers (provided in Table S1) to amplify bisulfite-converted DNA at these loci. Due to other CpGs included in the amplicons, a total of 124 CpGs were quantified. We analyzed livers from mice at E18.5 (*n* = 6), P1 (*n* = 5), P5 (*n* = 5), P10 (*n* = 6), P15 (*n* = 6), P20 (*n* = 6), and 9 wk old (*n* = 5). The amplification primers included 5′-end tails that allowed the addition of adapter sequences and barcodes for massively parallel sequencing by a second PCR. We then combined samples together and sequenced all amplicons simultaneously on an Illumina MiSeq to generate paired-end reads of 250 bp. The samples sequenced included both male and female animals, which were analyzed together, as we detected no differences in DNA methylation between sexes at these loci.

### Sequence data analysis

We checked sequence quality using FastQC (v0.9.0, Babraham Institute) and removed any reads containing adapter or PhiX sequences. We analyzed RRBS and LSBS data by aligning fastq files to the mouse genome (mm9) using Bismark (v0.7.6) with bowtie2, 20 processors, output as bam, and the default settings for all other options (Krueger and Andrews 2011). Bisulfite conversion rates were estimated using Bismark. We then combined data generated for each sample by different sequencing runs and summarized the DNA methylation data using the Bismark methylation_extractor. We quantified methylation for each CpG using perl scripts and imported the data into *R* (v3.2.1) for statistical analysis. For each sample, any CpG with fewer than 10 × coverage was considered as missing data. We removed any CpGs where more than 20% of samples had missing data and any CpG that was unmethylated (< 10%) in all samples of all ages. To identify samples with unusual missing data, we first determined CpGs that were most shared across samples by calculating the percentage of samples with data for each CpG. We then multiplied this weighting factor by 1 (read coverage ≥ 10) or 0 (missing data) for each CpG in each sample. If the sum of these values was less than the arbitrary cutoff of 50,000, the sample was removed from the analysis. This approach avoids removal of samples with relatively few CpGs covered that are shared by all other samples. It also avoids the inclusion of samples covering many CpGs that are not shared by other samples. One sample was thus removed from the time-course dataset (out of 90). Twelve additional samples produced no reads. We also performed a MDS analysis (Figure S1) to identify clear outliers and further removed three samples. Overall, 15 samples were analyzed in the discovery dataset and 74 in the time-course dataset.

For the LSBS data, we discarded any sequences mapping to a region that was not targeted and any CpG with < 100 × coverage or for which more than 20% of samples had missing data. We calculated the percent methylation at each locus for each sample and compared values between groups using a Student’s *t*-test. We derived false discovery rates (FDR) from *t*-test p-values using the qvalue package (v1.36.0) and generated plots using the ggplot2 package (Wickham 2009).

For the time-course RRBS experiment, we restricted our analyses of the timing of changes in DNA methylation to the CpGs that were identified as significantly differently methylated in the discovery RRBS dataset. We then calculated the change in methylation between consecutive time points and quantified the number of CpGs changing their methylation by more than 5 and 30%.

### Genomic feature analysis

We defined the genomic context for all CpGs assayed in our RRBS experiments using the following UCSC Genome browser tracks: DNase hypersensitivity from eight-week-old (GEO# GSM1014195, replicate #1) and E14.5 (GEO# GSM1014183) C57BL/6 liver (ENCODE/University of Washington data, peaks were combined using Bedtools), CpG islands, genic context [UCSC genes: exons, introns, and promoters (1 kb upstream of transcription start site)], CTCF binding sites (LICR TFBS Liver GEO# GSM918715), and 28-way conservation and histone modifications (LICR liver Histone data GEO# GSM1000140, GSM1000113, GSM1000150, GSM1000151, GSM769015, GSM1000111, GSM769014, GSM1000110, GSM1000152, and GSM1000153) (Rosenbloom *et al.* 2013). We calculated CpG density and GC content using 200 bp up- and downstream of each CpG assayed by RRBS. Promoter, exon, and intron tracks were made exclusive of each other by subtracting promoters from exons, and subtracting the remaining exons from the introns. We calculated the distance to each genomic feature for each CpG in the RRBS data using bedtools (Quinlan and Hall 2010).

We grouped CpGs according to the pattern of the DNA methylation change. We first analyzed all CpGs with significant change in DNA methylation (change in methylation ≥ 5% and FDR ≤ 0.1) and divided them according to the direction of the DNA methylation changes (*i.e.*, increased or decreased DNA methylation with age). We further subdivided these CpGs into discrete changes (50% or more of the total methylation change occurs between two consecutive time points), continuous changes (< 50% methylation changes between two consecutive time points but continuous increase or decrease with time), and other (all other changing CpGs).

We also calculated whether CpGs changing their DNA methylation occurred preferentially in specific genomic contexts by comparing their enrichment to 1000 random subsamplings of CpGs exactly matching the local CpG density. We then generated a p-value for each enrichment by quantifying how many subsamples had a greater number of CpGs in each feature than the observed data.

### Animal experimentation

All animals were handled and housed in accordance with standard NIH practices. All protocols were approved by the Institutional Animal Use and Care Committee.

### Data availability

All data used in this study are available in the NCBI GEO database under accession numbers GSE58129 and GSE79775. The authors state that all data necessary for confirming the conclusions presented in the article are represented fully within the article.

## Results

### Bisulfite sequencing data result summary

We generated two DNA methylation datasets using RRBS. We extensively sequenced liver samples from two time points for our discovery dataset to identify which CpGs changed methylation between E18.5 (*n* = 5) and 9 wk of age (*n* = 10). For this dataset, we generated between 6.5 and 83.6 million reads per sample (mean = 22.2 million, with bisulfite conversion rates > 99% for all samples) (Table S2) and were able to analyze 588,691 CpGs distributed throughout the genome. Each of these CpGs was covered by at least 10 reads in more than 80% of the samples. We removed any CpG that was unmethylated (< 10% methylation) in all samples, leaving 271,325 CpGs for further analyses. Note that removing CpGs that were never methylated from the analysis removed differences in local CpG density and GC content between CpGs that significantly changed their DNA methylation between E18.5 and 9 wk and those who remained unchanged (Figure S2). On average, the 271,325 CpGs analyzed were covered by 40 reads in each sample (Table S2). While this coverage is comparable to or greater than that typically used in epigenetic studies (Gifford *et al.* 2013; Xie *et al.* 2013; Kobayashi *et al.* 2013; Ziller *et al.* 2013; Liu *et al.* 2014; Fritz *et al.* 2013; Varley *et al.* 2013), it is important to note that this level of coverage only allows quantification of the percent methylation at a given CpG with modest accuracy: DNA methylation at a CpG covered by 40 reads can only be quantified in 2.5% intervals. Therefore, we only considered CpGs as significantly changed between age groups if, in addition to being statistically significant (FDR ≤ 0.1), the average difference in percent DNA methylation between groups was > 5% (*e.g.*, 5% methylation at E18.5 and 10% at 9 wk). In a similar dataset, we previously estimated that such analyses had 80% power to detect an 11% difference in DNA methylation (Cannon *et al.* 2014).

To validate the RRBS data, we performed LSBS at 11 loci (targeting 21 CpGs from the RRBS data and containing 124 CpGs total) randomly chosen from our discovery RRBS data, and analyzed 39 samples (E18.5 *n* = 6, P1 *n* = 5, P5 *n* = 5, P10 *n* = 6, P15 *n* = 6, P20 *n* = 6, and 9 wk *n* = 5). We generated 7 million reads resulting in an average of 178,375 reads per sample and a mean coverage of 2519 × per CpG. This experiment confirmed the results of the RRBS experiment (Figure S3) with no CpGs differing statistically in their methylation status as estimated by LSBS or RRBS (p > 0.05).

We also evaluated possible DNA methylation at non-CpG sites (CpH) in the discovery RRBS dataset. Out of 1,581,309 CpHs analyzed, only 356 (0.0002%) displayed DNA methylation > 5% for all samples in at least one group. At many of these sites, inspection of the aligned reads showed that the base following the C was a G in the sequenced sample but not in the reference (due to a SNP, an error in the reference genome sequence, or a mismapping of the read), leading to the incorrect classification of these CpGs as CpHs. Given the small number of putative methylated CpHs and the observation that many of these were likely misclassified, we focused the rest of our analyses on CpG methylation.

To identify the timing of DNA methylation changes in liver and determine if similar changes occur in other tissues, we generated a second RRBS (“time-course”) dataset. We analyzed liver, heart, and muscle samples from multiple time points: E18.5 (*n* = 4, 5, and 4, respectively), P1 (*n* = 4, 4, and 5), P5 (*n* = 4, 4, and 3), P10 (*n* = 3, 4, and 4), P15 (*n* = 3, 5, and 4), and P20 (*n* = 5, 5, and 4). Note that, since we combined more samples per sequencing lane, this dataset has a lower coverage than the discovery dataset: we generated between 4.5 million and 44.9 million reads per sample (with bisulfite conversion rates > 99% for all samples) and, after filtering, were able to analyze 70,259 CpGs across six time points in the liver samples, 53,520 CpGs in heart samples, and 46,085 CpGs in muscle samples (Table S2). The average coverage was 50.4, 45.7, and 46.3 for liver, heart, and muscle, respectively.

### RRBS reveals extensive postnatal changes in liver methylation

To identify outliers and preliminarily assess parameters influencing variation in global DNA methylation, we performed a MDS analysis using the entire RRBS dataset. In both the discovery and time-course datasets, the liver samples clustered according to the age of the animal (Figure S4). There were minor differences between the methylation patterns of the E18.5 samples generated in the discovery and time-course experiments (Figure S4) that could be possibly due to biological differences (*e.g.*, different mouse cohort or uncertainty in timed mating) or technical factors. However, it is important to note that all analyses were conducted within each cohort and that the samples of each cohort were randomized and processed identically at the same time.

**Figure 1:**
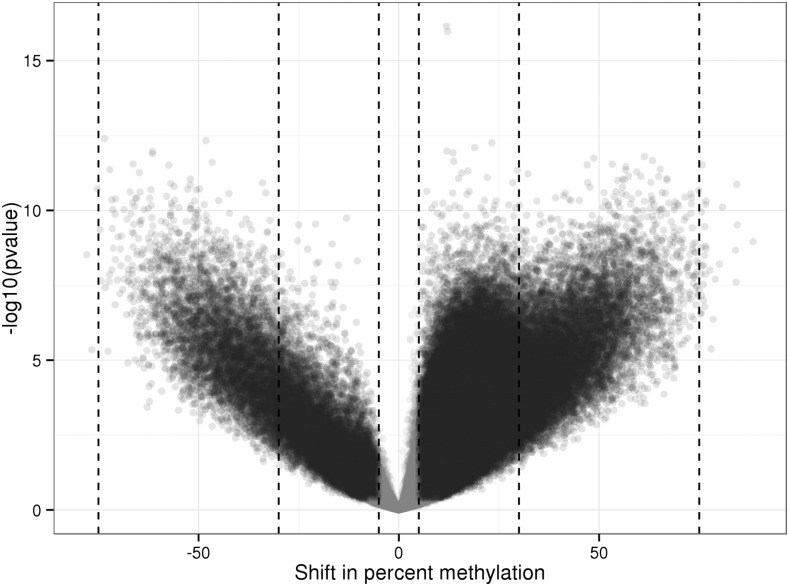
Genome-wide changes in DNA methylation in the liver after birth. There are numerous differences in DNA methylation between E18.5 and 9 wk old mice. Each point represents one of the 271,325 CpGs analyzed by RRBS and is displayed according to the magnitude of the change in DNA methylation (*x*-axis, in %) and statistical significance (*y*-axis, in –log10 of the p-value). Fifty on the *x*-axis represents an increase in DNA methylation of 50% points (*e.g.*, from 5 to 55% methylation) as the animals age. Black points indicate CpGs that changed their methylation by more than 5% and an FDR ≤ 0.1. Gray points are CpGs whose methylation level was not significantly changed. Vertical dotted lines are at 5, 30, and 75% gain/loss of methylation. E, embryonic day; FDR, false discovery rate; RRBS, Reduced Representation Bisulfite Sequencing.

We specifically tested whether each CpG in the discovery dataset significantly changed its methylation status between E18.5 and 9 wk. We identified a total of 214,709 CpGs (out of 271,325 or 79%) that significantly changed their DNA methylation by more than 5% (Figure 1, FDR ≤ 0.1). Of these, 18,863 CpGs changed by more than 30%.

We detected both gain and loss of DNA methylation between E18.5 and 9 wk but, overall, the distribution was biased toward increased DNA methylation with age: 89% of the significantly changed CpGs were more methylated in adult mice (the bias was similar when restricting the analysis to CpGs that changed the DNA methylation by more than 30%). The magnitude of the DNA methylation changes varied extensively among CpGs, with the methylation status of some CpGs changing by up to 88% (Figure 1).

To determine if the age-associated changes in DNA methylation were unique to liver, we used the time-course RRBS dataset to compare the patterns in liver, heart, and muscle samples. MDS analysis showed that the clustering by age was most obvious in the liver, and that the dimension separating the samples according to the age of the animals explained a larger proportion of the variance in the liver than in the heart and muscle (20.2% *vs.* 9.0% and 9.6%, respectively) (Figure S4). We also tested specifically for differences in DNA methylation at each CpG between E18 and P20 after randomly subsampling each tissue and age group to *n* = 3 for identical comparisons. In these lower power but comparable analyses, we found that 6998 CpGs out of 70,259 (9.9%) changed their DNA methylation by > 5% in the liver (FDR ≤ 0.1), while only 8 of 53,520 CpGs were changed in heart and no CpGs, out of 46,085, changed in muscle. Overall, these analyses showed that there are extensive DNA methylation changes in liver after birth and that this pattern is not observed in other tissues analyzed, suggesting that it is not an organism-wide phenomenon.

### Epigenetic changes occur predominately after postnatal day five

**Figure 2:**
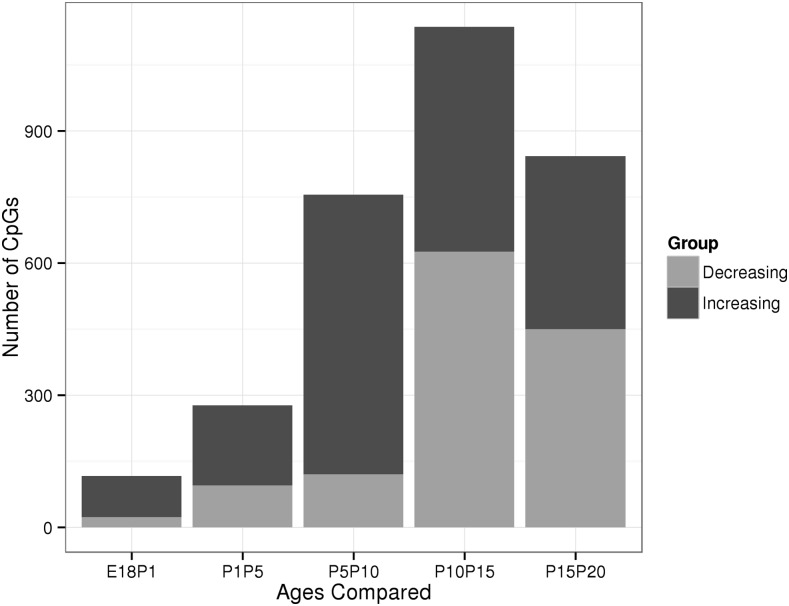
Count of CpGs changing by 30% or more between adjacent time points. We counted CpGs that changed by 30% or more (*e.g.*, from 30 to 60% methylation) between adjacent time points. The direction of change is indicated by color. The most changes occurred between P10 and P15. E, embryonic day; P, postnatal day.

To define the timing of epigenetic changes, we analyzed the CpGs that were significantly changed in the discovery dataset in an independent set of liver samples from six time points between E18.5 and P20. Data from the two datasets were highly concordant, with 93.2% of the CpGs with significant change in DNA methylation in the time-course dataset also significantly changed in the discovery dataset (Figure S5). However, due to the lower sequence coverage in the time-course data, only 53,682 CpGs were present in both analyses and, overall, we were able to follow the temporal changes in DNA methylation at 42,889 CpGs that significantly changed their methylation status in the discovery dataset (note that, due to the smaller sample size, only 11,942 of those CpGs reached statistical significance in the time-course dataset). We first calculated the number of CpGs that changed their DNA methylation between consecutive time points to identify when most changes occur. When considering only the CpGs that changed their methylation by > 30% between two ages, we observed that most DNA methylation changes occurred after P5 (Figure 2). Changes in DNA methylation at many CpGs were sustained over several days, confirming that these changes were genuine and not statistical artifacts (Table S3). For example, out of 1136 CpGs changing their methylation by more than 30% between P10 and P15, 223 also changed between P15 and P20 (while only five had changed between E18.5 and P1) (Table S3).

Overall our analyses revealed extensive and sustained epigenetic changes in the liver after birth, with the majority of these changes occurring between day 5 and day 20.

### Variation in cellular division rates leaves its footprint in the DNA methylation patterns but does not explain the epigenetic changes associated with age

**Figure 3:**
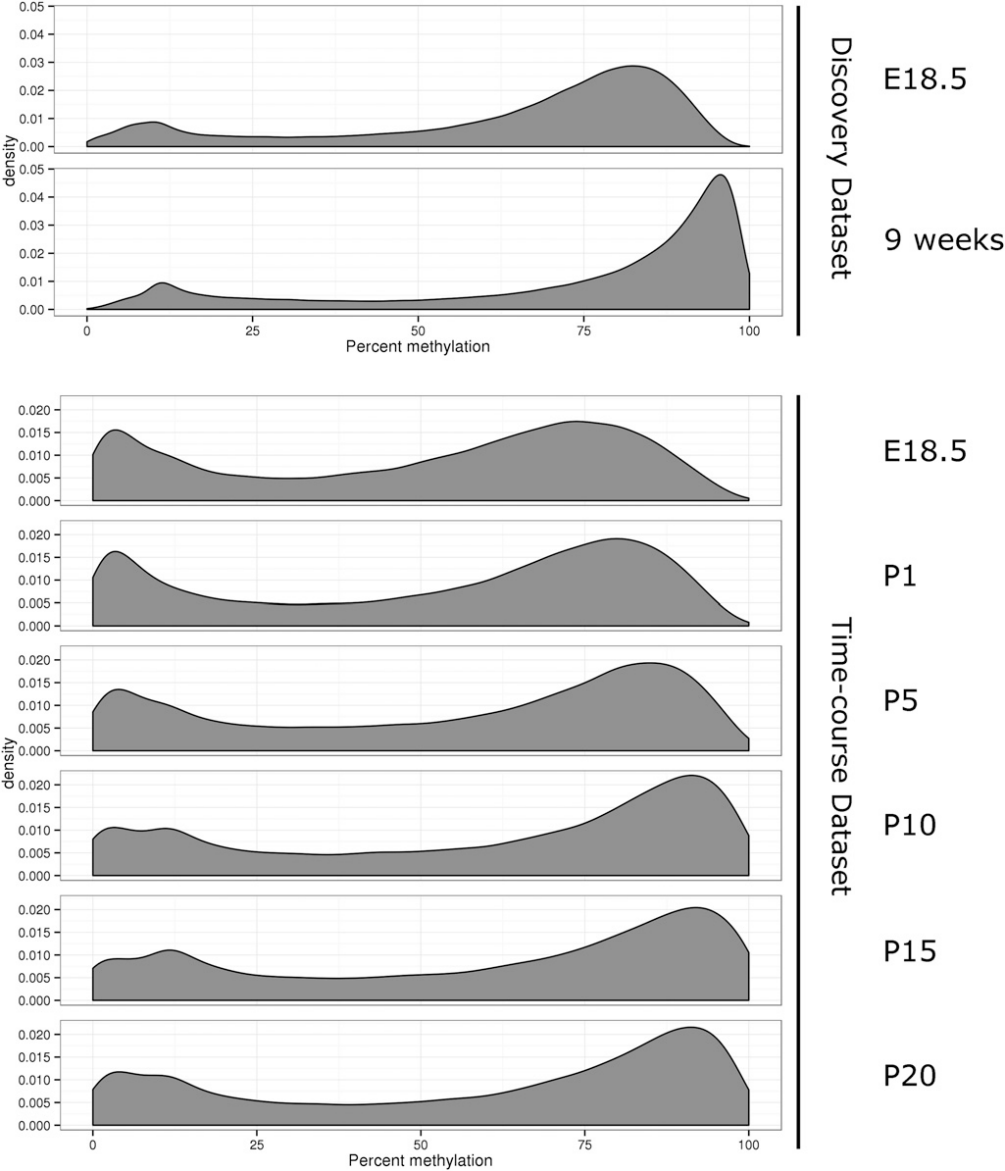
Distribution of the extent of DNA methylation throughout the genome for each age group. Overall genomic methylation patterns change with age. The figure shows the proportion of CpGs (*y*-axis) with a specific methylation level (*x*-axis in percent) for the discovery and time-course datasets. Note that the maximum methylation level increases with age, possibly reflecting the decrease in cell divisions (see main text for details). E, embryonic day; P, postnatal day.

The liver grows dramatically during the early postnatal period, weighing over five times more at P20 than at birth (Figure S6). Extensive cell division could affect the DNA methylation patterns of a given organ, since the newly synthesized DNA molecules initially lack DNA methylation and are hemimethylated (Hervouet *et al.* 2012; Desjobert *et al.* 2015). However, this mechanism is unlikely to explain (i) the loss of DNA methylation observed at 11% of the changing CpGs and (ii) the magnitude of the changes in DNA methylation observed at many CpGs. Interestingly, we noted that, when comparing the same CpGs across samples, the maximum DNA methylation levels observed in our RRBS experiments differed according to the age of the animal (Figure 3) and were consistent with more cellular divisions occurring in newborn liver than adult liver. This is unlikely to be due to batch effects, as the same pattern was observed in both the discovery and time-course datasets. These changes in cellular division rates inferred from the DNA methylation patterns were corroborated by analyses of *Cyclin* gene expression: the *Cyclins A2*, *B1*, *E1*, and *E2* showed a continuous decrease in gene expression from E18.5 to P20 and were barely detectable afterward (Figure S7).

Since cell division cannot explain the extensive changes in DNA methylation observed in liver postnatally, we then investigated the possible contribution of variations in cell composition.

### HSC migration precedes epigenetic changes

Prior to birth, the liver is the primary site of hematopoiesis and HSCs migrate to the bone marrow just before or after birth (Mikkola and Orkin 2006). Histological analyses showed that the liver samples consisted of more than 60% HSCs at birth, but that HSCs made up < 4% of the livers by P10 (Table 1). Therefore, the migration of HSCs from the liver postnatally is not sufficient to explain the dramatic epigenetic changes that we observed between P10 and P20. Note that our data are in agreement with previously published data (Waterland *et al.* 2009), although we observed a slightly higher proportion of remaining HSCs in our experiments at P5 than previously reported. After P10, the livers were composed primarily of hepatocytes and hepatocyte precursors, with adult-like morphology (easily discernible lobules with a central vein and portal triads) and cellular composition (primarily hepatocytes). Figure S8 shows representative H&E stained liver sections from the different age groups.

**Table 1:** Tissue composition of the liver from E18.5 through P20

Age	Liver Parenchyma (%)	Hematopoietic Cells (%)
E18.5	52	48
P1	38	62
P5	56	38
P10	96	4
P15	99	1
P20	100	0

E, embryonic day; P postnatal day.

### Postnatal epigenetic reprogramming occurs concurrently with hepatocyte differentiation

**Figure 4:**
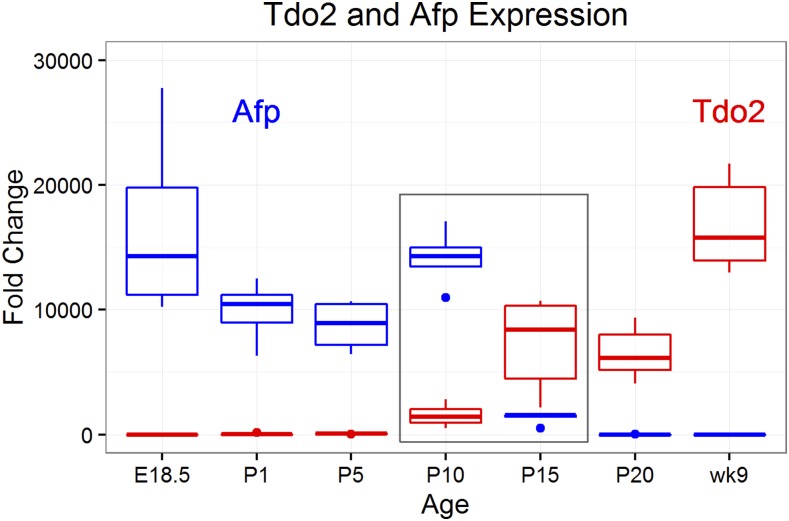
Timing of terminal differentiation of hepatocytes. We quantified *Afp* (a marker of hepatocyte precursors) and *Tdo2* (a marker of differentiated hepatocytes) using qRT-PCR in liver at ages from E18.5 through 9 wk. *Afp* decreased dramatically between P10 and P15 (boxed), which was the same time period during which a large increase in *Tdo2* occurred. E, embryonic day; P, postnatal day; qRT-PCR, quantitative reverse transcription-polymerase chain reaction.

To characterize the timing of hepatocyte differentiation, we quantified the abundance of transcripts specific to a given cell type in each liver sample. *Albumin* expression, a marker of hepatocytes and hepatocyte precursors (Tanimizu and Miyajima 2004), decreased between E18.5 and P5. *Albumin* expression then increased to roughly twice the E18.5 value at P10 and remained stable thereafter (Figure S7). These observations mirrored our histological results, which showed a decrease in the proportion of hepatocytes at P1 followed by a large increase in hepatocytes between P5 and P10 (Table 1). *Tryptophan 2,3-dioxygenase* (*Tdo2*) (Cai *et al.* 2007; Nagao *et al.* 1986) and *serine hydratase* (*Sds*) (Noda *et al.* 1990) are markers of differentiated hepatocytes, and *alpha-fetoprotein* (*Afp*, Figure 4) (Tanaka *et al.* 2009) is a marker of hepatocyte precursors. Analysis of these markers revealed that hepatocyte differentiation began at P5 with the largest change occurring between P10 and P15 (Figure 4 and Figure S7). These observations indicate that the extensive epigenetic changes observed in the liver after birth largely overlap with the timing of the terminal differentiation of hepatoblasts into hepatocytes.

### Epigenetic reprogramming during hepatocyte differentiation localizes in enhancer elements

To investigate the biological context of these epigenetic changes, we tested whether specific genic or genomic features were associated with these extensive postnatal DNA methylation changes. For these analyses, we considered separately CpGs that changed by between 5 and 30%, or those that changed by a minimum of 30%. We analyzed CpGs grouped by the pattern and direction of the change in DNA methylation (as outlined in the *Materials and Methods*).

In our dataset, out of the 2293 CpGs located in promoter regions (defined as 1 kb upstream of a transcription start site), only 5.3% changed their DNA methylation by more than 30%. Compared to the percent of all CpGs analyzed that changed their methylation by more than 30% (8.5%), this represents a 1.60-fold depletion, suggesting that DNA methylation at CpGs in promoter regions is proportionally less often affected by the age of the animal than an average CpG in the genome. However, this analysis does not take into account possible differences in CpG content between different genomic contexts that may affect DNA methylation. Therefore, we calculated the enrichment in differentially methylated CpGs of each genomic feature by randomly subsampling regions of the genome with similar CpG density (see *Materials and Methods* for details).

**Figure 5:**
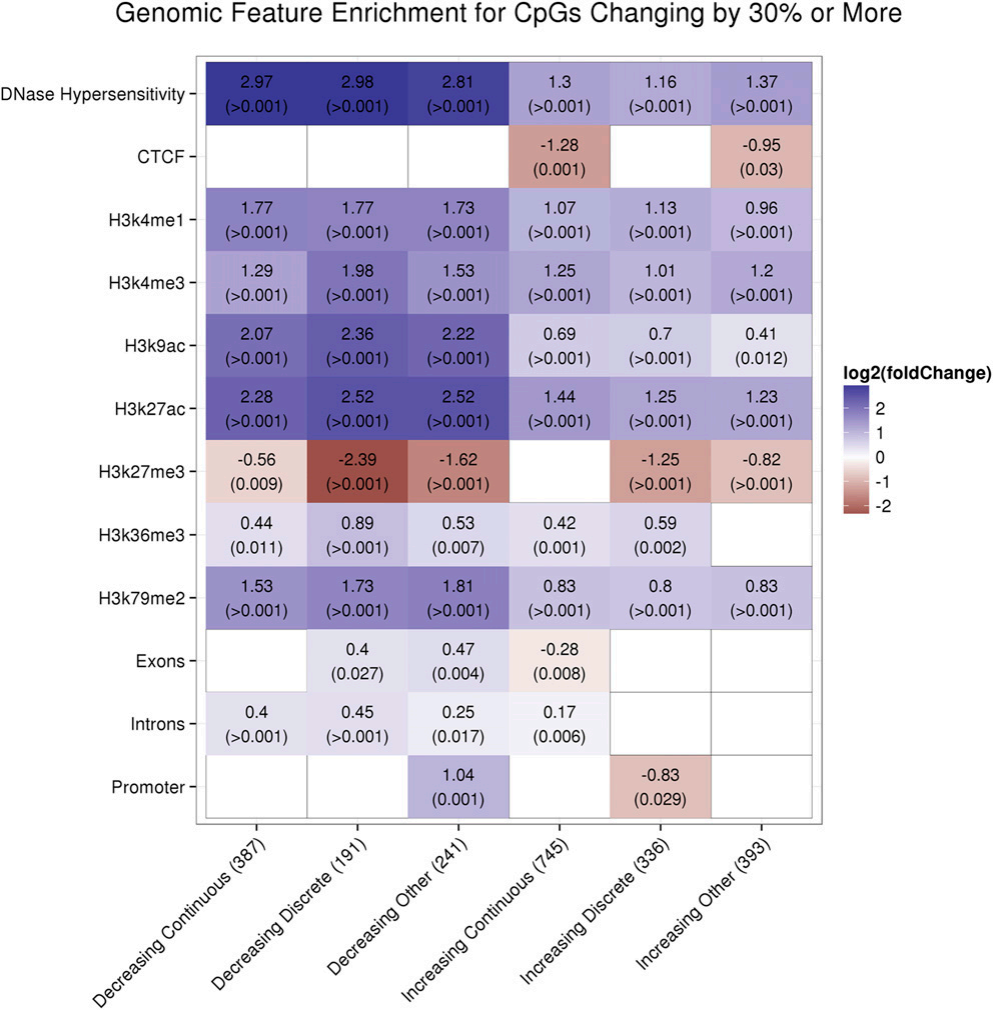
Enrichment for CpGs changing by 30% or more in different genomic contexts. We calculated enrichment values for genomic contexts for CpGs changing by 30% or more. The CpGs were grouped by direction and pattern of change as described in the *Materials and Methods*. Note that only statistically significant enrichment values are shown. We generated p-values, indicated in parentheses beneath the fold change values, for each by resampling the data 1000 times, while correcting for local CpG density as detailed in the *Materials and Methods*.

After this adjustment, we observed that genic context (promoters, exons, and introns) had little influence on differential methylation between ages. By contrast, temporal changes in DNA methylation occurred much more often than expected by chance in regions of DNase hypersensitivity (2.34–7.89-fold enrichment), with marks commonly found in enhancers such as H3K4me1 (1.95–3.41-fold enrichment), or H3K27ac (2.35–5.74-fold enrichment) (Figure 5). In fact, despite only roughly 25% of the CpGs in our dataset overlapping H3K4me1 regions, more than 85% of the CpGs that decreased methylation by > 30% overlapped H3K4me1 marks. For CpGs that increased by > 30%, 50–57% of CpGs overlapped H3K4me1 marks. Interestingly, H3K4me3 was enriched (2.01–3.94-fold enrichment) and H3K27me3 was depleted for differential methylation (1.47–5.24-fold depletion), while both histone marks are typically found in promoters, though recent evidence suggests that H3K4me3 may also be present in select enhancers (Chen *et al.* 2015; Pekowska *et al.* 2011). H3K9ac, which is generally associated with transcriptionally active chromatin (Qiao *et al.* 2015), was also enriched for differential methylation (1.33–5.13-fold enrichment). CTCF binding sites did not seem preferentially affected by the changes in DNA methylation (1.93–2.43-fold depletion). H3K36me3, often found toward the 3′-end of actively transcribed genes (Wagner and Carpenter 2012), was slightly enriched (1.34–3.51-fold enrichment), except for CpGs that increased in the “other” pattern group. H3K79me2, which is generally associated with transcriptionally active genes (Steger *et al.* 2008), was slightly enriched (1.74–3.51-fold enrichment). The detailed analyses for all CpGs that changed by 5–30% are presented in Figure S9. Overall, our analyses revealed that most of the age-related epigenetic changes occur largely independently of the genic context and primarily in regions where histone marks are suggestive of the presence of enhancers (Onder *et al.* 2012; Guenther *et al.* 2008; Yoo and Hennighausen 2012; Rada-Iglesias *et al.* 2011; Zentner *et al.* 2011; Hon *et al.* 2013).

## Discussion

**Figure 6:**
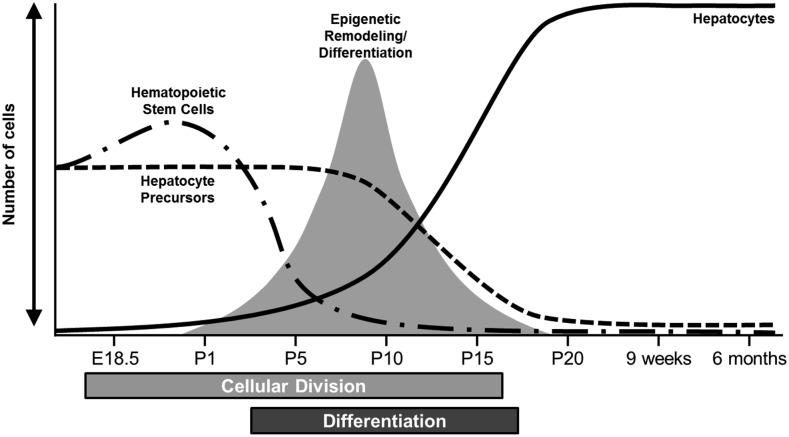
Summary of the changes occurring in postnatal liver. The figure summarizes the cellular changes occurring in the liver after birth including: the decrease in hematopoietic cell population (dash/dot line), the decrease in the number of hepatocyte precursor cells (dashed line), and the increase in the number of hepatocytes (solid line). The figure also shows when most of the epigenetic remodeling occurs. E, embryonic day; P, postnatal day.

In contrast to histone modifications, DNA methylation marks are typically considered to be established early in development and to be stable during adult life (Bird 2002; Law and Jacobsen 2010). However, recent *in vitro* studies have shown that DNA methylation can change during terminal differentiation of cells (Stadler *et al.* 2011; Scharer *et al.* 2013; Laurent *et al.* 2010). We showed here that extensive DNA methylation changes occur postnatally in the liver, mostly between P5 and P20. Based on our analysis, HSCs constitute more than half the cells of the liver at E18.5, but only represent ∼4% of the cells in the liver at P10. This indicates that the extensive changes in DNA methylation observed in postnatal liver are unlikely to be caused by the exodus of HSC from the liver. The liver also dramatically increases in size after birth, primarily due to cell division and multiplication of hepatocytes. Cell division can affect estimation of DNA methylation since, after DNA replication, there is a short period when the newly synthesized DNA strand is unmethylated. Thus, if 10% of the cells of a given sample are actively dividing, one would expect a decrease of DNA methylation of 5% (assuming that all cells are methylated at this locus). While this temporary loss of methylation would affect the entire genome, it is possible that methylation marks in some regions may be systematically restored faster than in other regions (based on the kinetics of DNA methyltransferase protein binding or the local chromatin structure). While our analyses of the DNA methylation patterns are consistent with variations in cell division rates, this cellular process is unlikely to explain the magnitude of the DNA methylation changes that we observe at many loci throughout the genome. By contrast, the terminal differentiation of hepatoblasts into hepatocytes coincides with the timing of the DNA methylation changes in the postnatal liver and suggests that this cellular process is responsible for the epigenetic changes in the liver (Figure 6). Therefore, analyses of the genomic context of the CpGs that are differentially methylated at different time points might provide important insights into the biological mechanisms responsible for terminal differentiation of hepatocytes. This knowledge is particularly valuable since we do not currently have a robust model to study this phenomenon *in vitro*. Our study reveals that most genome-wide changes in DNA methylation occurred outside of genes and gene promoters, which also highlights the advantage of a global approach rather than studies that focus almost exclusively on gene promoters (Waterland *et al.* 2009). In our study, differentially methylated CpGs were significantly overrepresented in regions where histone marks (H3K27ac and H3K4me1) indicate the presence of distal gene enhancers (see *e.g.*, Figure S10). In particular, these regulatory regions were highly enriched in CpGs that decreased their methylation status from E18.5 through 9 wk. These findings agree with previous studies that showed the critical role of enhancer regions in cellular differentiation, and suggests that the epigenetic mechanisms controlling embryonic stem cell fate are similar to those underlying terminal differentiation (Gifford *et al.* 2013; Xie *et al.* 2013; Whyte *et al.* 2012; Kaaij *et al.* 2013). Our study also provides a solid foundation for future studies aimed at understanding which genes these differentially methylated enhancers regulate, and how these genes drive the differentiation of hepatoblasts.

Our findings have implications for diseases of the liver. First, our study describes extensive epigenetic alterations that occur during early postnatal development, a critical period for the establishment of adult liver function. While we cannot definitively explain the cause of these epigenetic changes (due to the cellular complexity of the developing liver), we believe, based on the timing of these changes, that they are primarily driven by the terminal differentiation of hepatocytes and speculate that DNA methylation is an important mechanism in cell fate determination in these cells. Further investigations of the molecular mechanisms underlying these DNA methylation changes could improve our understanding of how hepatocytes terminally differentiate, which will be critical to better understand the regenerative properties of the liver and explore possible therapeutic avenues exploiting this feature. Second, our analyses showed that DNA methylation in the liver is dramatically altered after birth. This observation is important as DNA methylation has been proposed to underlie the translation of maternal stimulus into long-lasting molecular consequences. The *in utero* environment has been shown in many animal models and epidemiological studies to influence the risk of developing diseases in adulthood (Barker 1997; Warner and Ozanne 2010). Our results suggest that further research is needed to determine if changes to DNA methylation *in utero* are maintained through the extensive reprogramming that coincides with hepatocyte differentiation and show if modification of DNA methylation in the liver could be responsible for this phenomenon.

Overall, our findings highlight the complexity and dynamicity of epigenetic regulation in the liver during early postnatal development, and point toward aspects of liver biology that are important for future research to investigate in order to better understand the mechanisms underlying the terminal differentiation of hepatocytes, as well as the molecular etiology of liver diseases.

## 

## Supplementary Material

Supplemental Material
