# Supplementary material for: Extensive Epigenetic Changes Accompany Terminal Differentiation of Mouse Hepatocytes After Birth
Source: G3 (Bethesda). 2016 Sep 21;6(11):3701–9. doi: 10.1534/g3.116.034785 (PMC5100869; doi:10.1534/g3.116.034785)
Supplement: Supplemental Material [file supp_6_11_3701__index.html]

Extensive Epigenetic Changes Accompany Terminal Differentiation of Mouse Hepatocytes After Birth — Supplemental Material 

# Extensive Epigenetic Changes Accompany Terminal Differentiation of Mouse Hepatocytes After Birth

## Supplemental Material for Cannon *et al.*, 2016

**Files in this Data Supplement:**

- Figure S1 - Multiple dimensional scaling analyses of outliers. (.pdf, 211 KB)
- Figure S10 - Example genome browser view of E18.5 and nine week methylation and histone modifications. (.pdf, 259 KB)
- Table S3 - Shared CpGs changing between time periods. (.pdf, 183 KB)
- Figure S2 - GC/CpG bias. (.pdf, 234 KB)
- Figure S3 - RRBS/LSBS comparison. (.pdf, 296 KB)
- Figure S4 - Multiple dimensional scaling analyses. (.pdf, 276 KB)
- Figure S5 - Comparison of discovery and time-course RRBS datasets. (.pdf, 206 KB)
- Figure S6 - Liver weight from P1 through P20. (.pdf, 158 KB)
- Figure S7 - Gene expression of *Cyclins* and hepatocyte expressed genes. (.pdf, 282 KB)
- Figure S8 - Liver histology. (.pdf, 395 KB)
- Figure S9 - Enrichment for CpGs changing by between 5% and 30% in different genomic contexts. (.pdf, 245 KB)
- Table S1 - PCR primers. (.xls, 48 KB)
- Table S2 - Sequence data summary numbers. (.xlsx, 16 KB)
