# Supplementary material for: Extensive Epigenetic Changes Accompany Terminal Differentiation of Mouse Hepatocytes After Birth
Source: G3 (Bethesda). 2016 Sep 21;6(11):3701–9. doi: 10.1534/g3.116.034785 (PMC5100869; doi:10.1534/g3.116.034785)
Supplement: Supplemental Material [file supp_g3.116.034785_FigureS1.pdf]

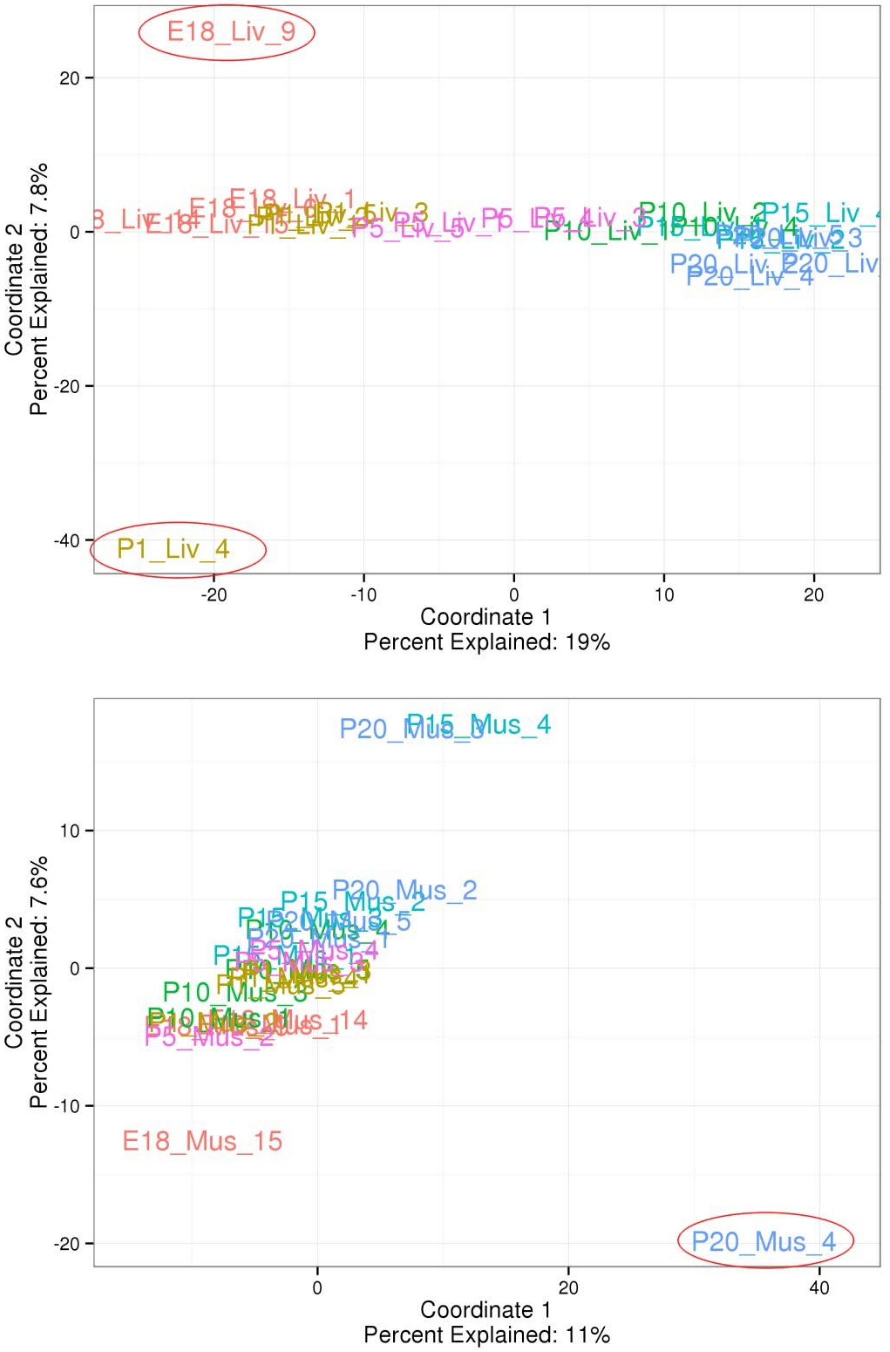


Figure S1: Multiple dimensional scaling analyses of outliers

We performed multiple dimensional scaling to identify outliers in our datasets. Coordinates 1 and 2 are presented as scatterplots with the age of each sample labeled. Outliers removed from the analyses are circled.
