# Supplementary material for: Extensive Epigenetic Changes Accompany Terminal Differentiation of Mouse Hepatocytes After Birth
Source: G3 (Bethesda). 2016 Sep 21;6(11):3701–9. doi: 10.1534/g3.116.034785 (PMC5100869; doi:10.1534/g3.116.034785)
Supplement: Supplemental Material [file supp_g3.116.034785_FigureS10.pdf]

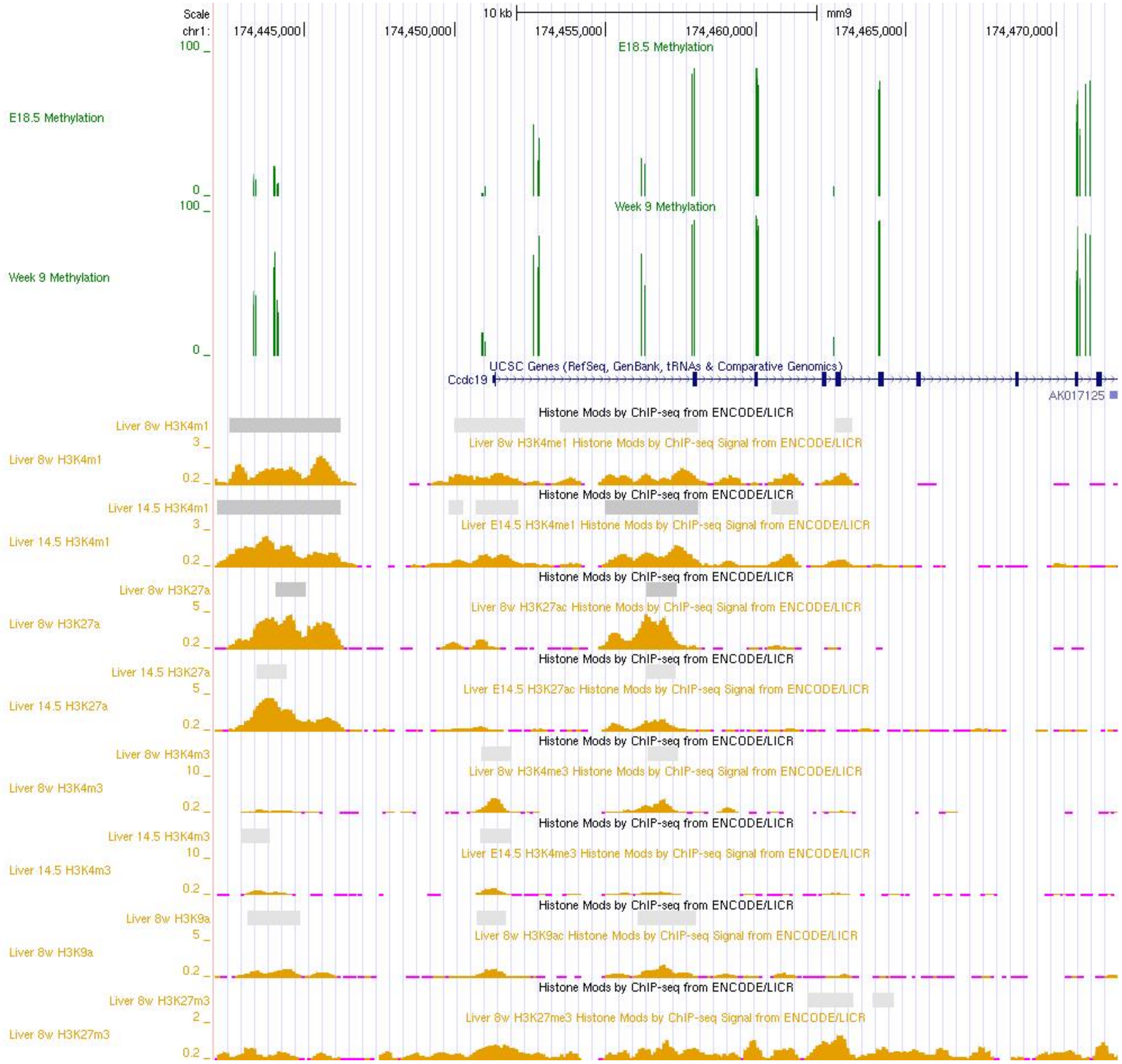

Figure S10: Example genome browser view of E18.5 and nine week methylation and histone modifications.

We uploaded average methylation data for E18.5 and nine week samples from the discovery dataset to the UCSC browser as bedGraph files. Encode histone modification data are shown below. DNA methylation changes most dramatically upstream of the gene in a region overlapping H3K4me1 and H3K27ac histone modifications. Methylation in the promoter and genic regions are less affected except for intronic methylation overlapping regions with histone modifications.
