# Supplementary material for: Extensive Epigenetic Changes Accompany Terminal Differentiation of Mouse Hepatocytes After Birth
Source: G3 (Bethesda). 2016 Sep 21;6(11):3701–9. doi: 10.1534/g3.116.034785 (PMC5100869; doi:10.1534/g3.116.034785)
Supplement: Supplemental Material [file supp_g3.116.034785_TableS3.pdf]

Table S3: Shared CpGs changing between time periods

	**E18-P1**	**P1-P5**	**P5-P10**	**P10-P15**	**P15-P20**
**E18-P1**	**117**	**2**	**6**	**5**	**2**
**P1-P5**		**277**	**24**	**20**	**7**
**P5-P10**			**754**	**103**	**52**
**P10-P15**				**1,136**	**223**
**P15-P20**					**843**
