# Supplementary material for: Extensive Epigenetic Changes Accompany Terminal Differentiation of Mouse Hepatocytes After Birth
Source: G3 (Bethesda). 2016 Sep 21;6(11):3701–9. doi: 10.1534/g3.116.034785 (PMC5100869; doi:10.1534/g3.116.034785)
Supplement: Supplemental Material [file supp_g3.116.034785_FigureS2.pdf]

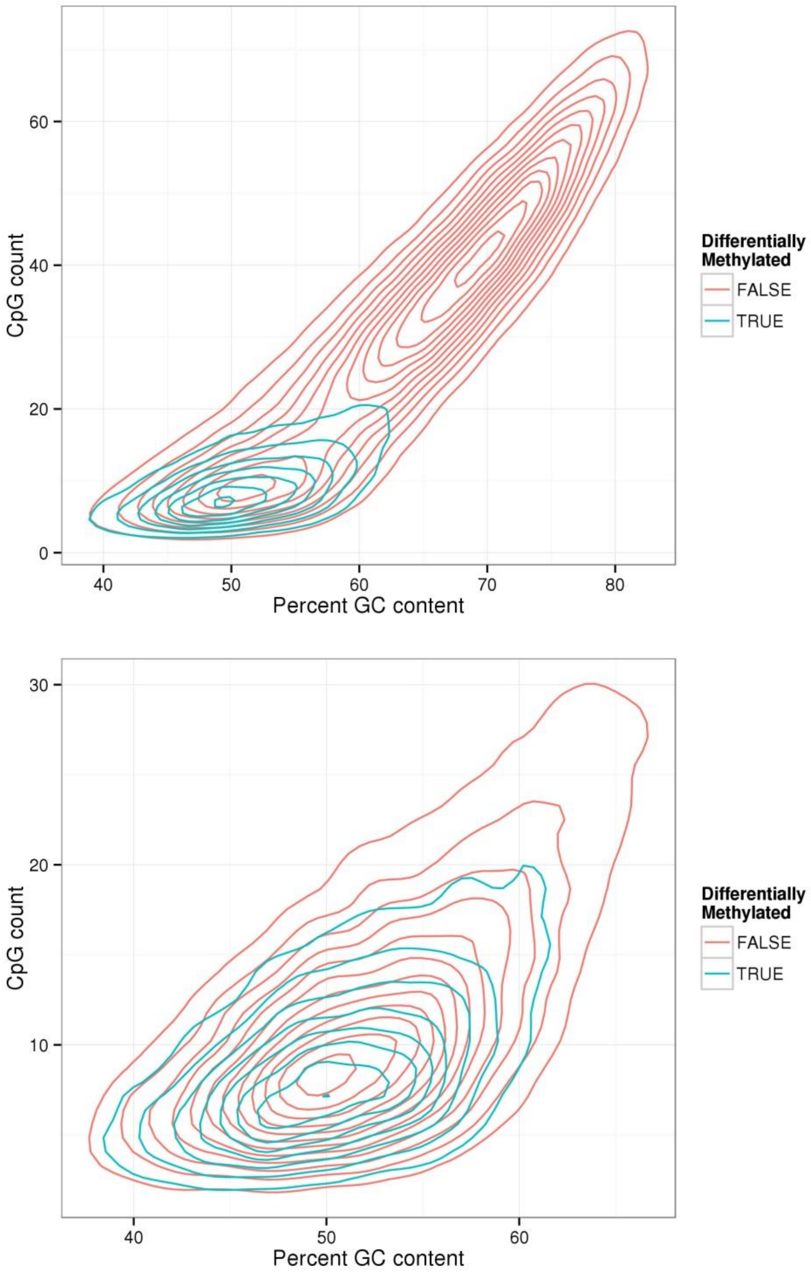

Figure S2: GC/CpG bias

Initially, there was a bias in the RRBS data due to differences in local GC and CpG counts in a large population of CpGs that are unmethylated in all samples (Top). After removal of all CpGs unmethylated in all samples, the bias was largely eliminated (bottom).
