# Supplementary material for: Extensive Epigenetic Changes Accompany Terminal Differentiation of Mouse Hepatocytes After Birth
Source: G3 (Bethesda). 2016 Sep 21;6(11):3701–9. doi: 10.1534/g3.116.034785 (PMC5100869; doi:10.1534/g3.116.034785)
Supplement: Supplemental Material [file supp_g3.116.034785_FigureS3.pdf]

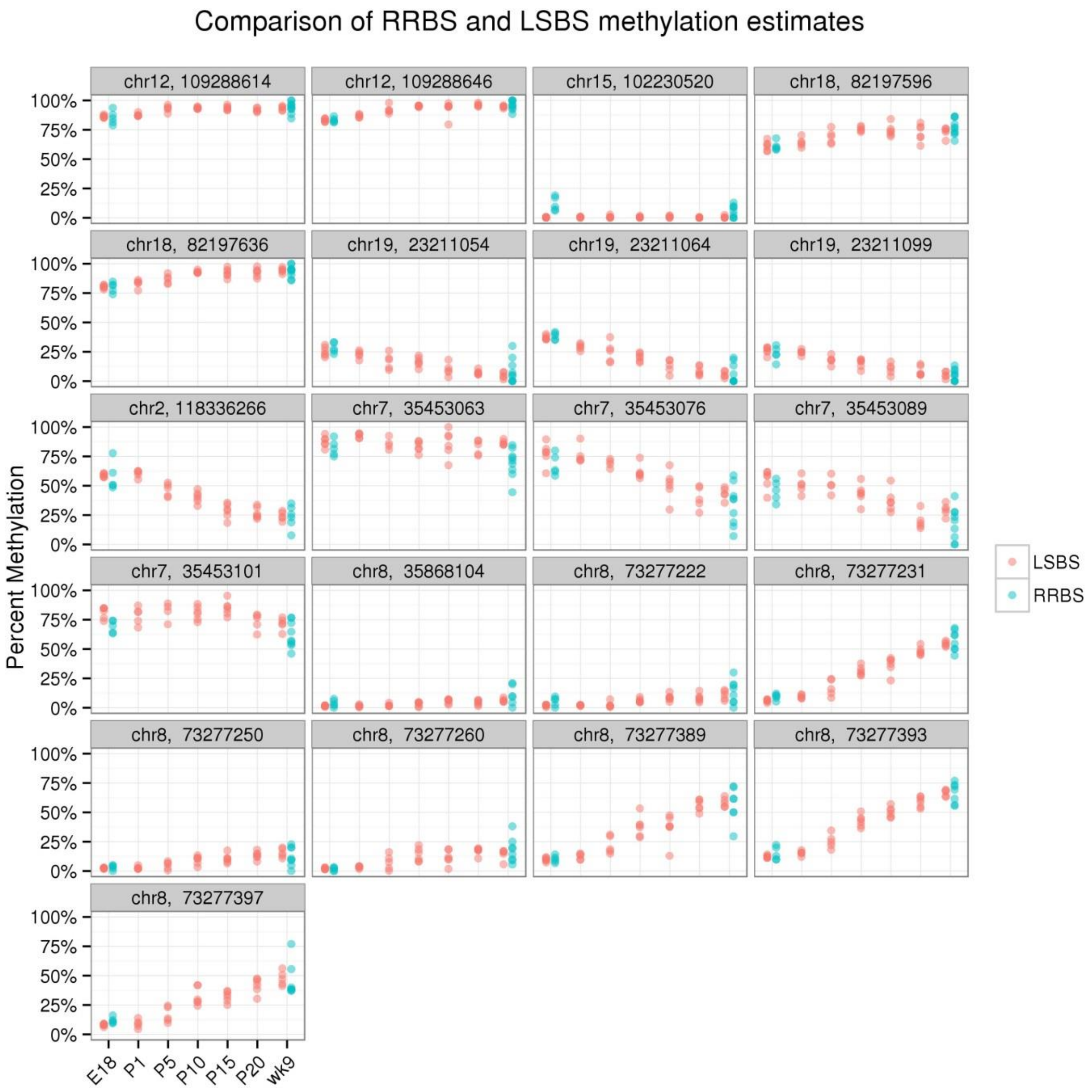

Figure S3: RRBS/LSBS comparison

To determine the reliability of the RRBS data (blue points), we generated LSBS data (red points) for CpGs covered by the RRBS data. No CpGs were significantly different between the two datasets ($p > 0.05$).
