# Supplementary material for: Extensive Epigenetic Changes Accompany Terminal Differentiation of Mouse Hepatocytes After Birth
Source: G3 (Bethesda). 2016 Sep 21;6(11):3701–9. doi: 10.1534/g3.116.034785 (PMC5100869; doi:10.1534/g3.116.034785)
Supplement: Supplemental Material [file supp_g3.116.034785_FigureS4.pdf]

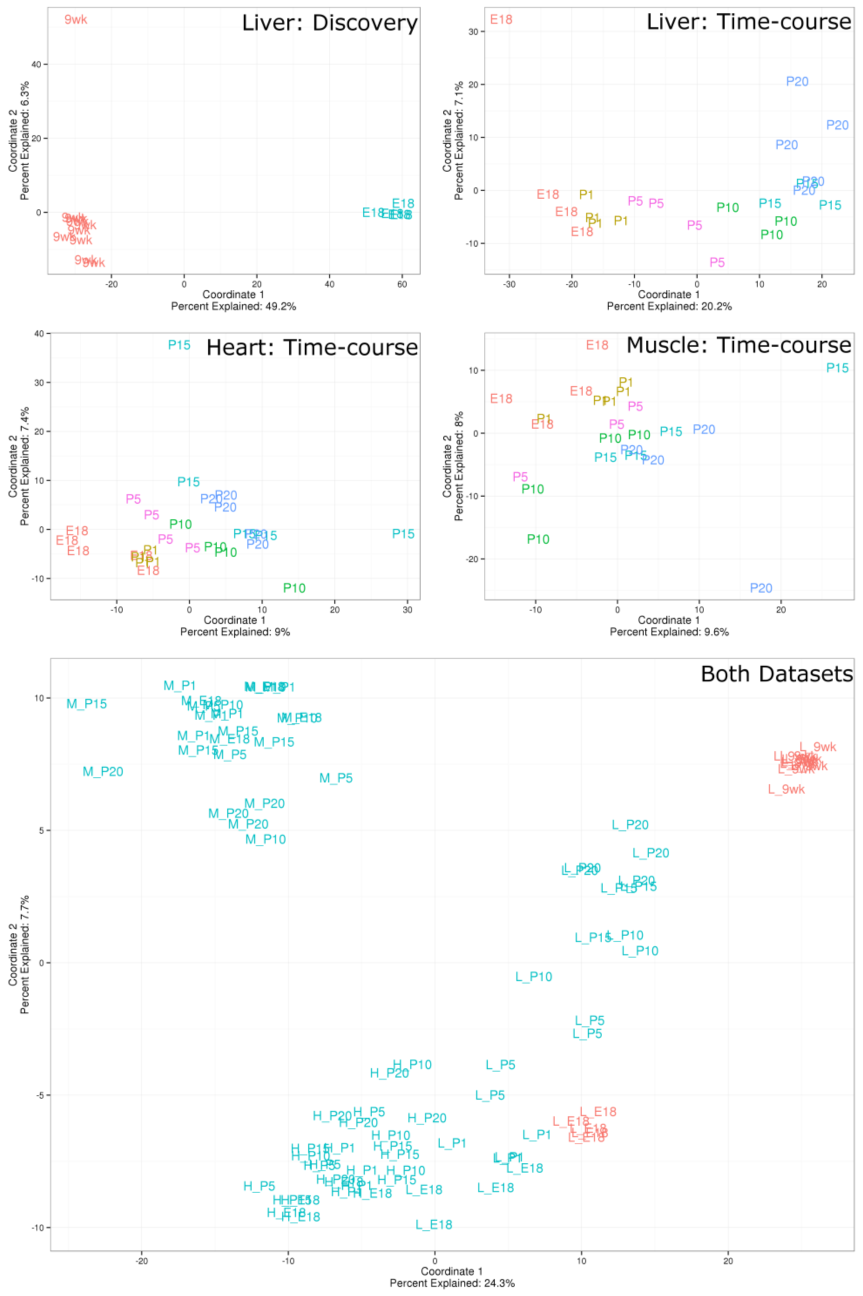

Figure S4: Multiple dimensional scaling analyses

To determine how age influenced methylation in the RRBS datasets, we performed multiple dimensional scaling analyses. Coordinates 1 and 2 are presented as scatterplots with the age of each sample labeled. Age separated the samples along the first dimension for all four datasets, thought the percent of the variance in the data explained by that vector was greatest for the liver samples.
