# Supplementary material for: Extensive Epigenetic Changes Accompany Terminal Differentiation of Mouse Hepatocytes After Birth
Source: G3 (Bethesda). 2016 Sep 21;6(11):3701–9. doi: 10.1534/g3.116.034785 (PMC5100869; doi:10.1534/g3.116.034785)
Supplement: Supplemental Material [file supp_g3.116.034785_FigureS5.pdf]

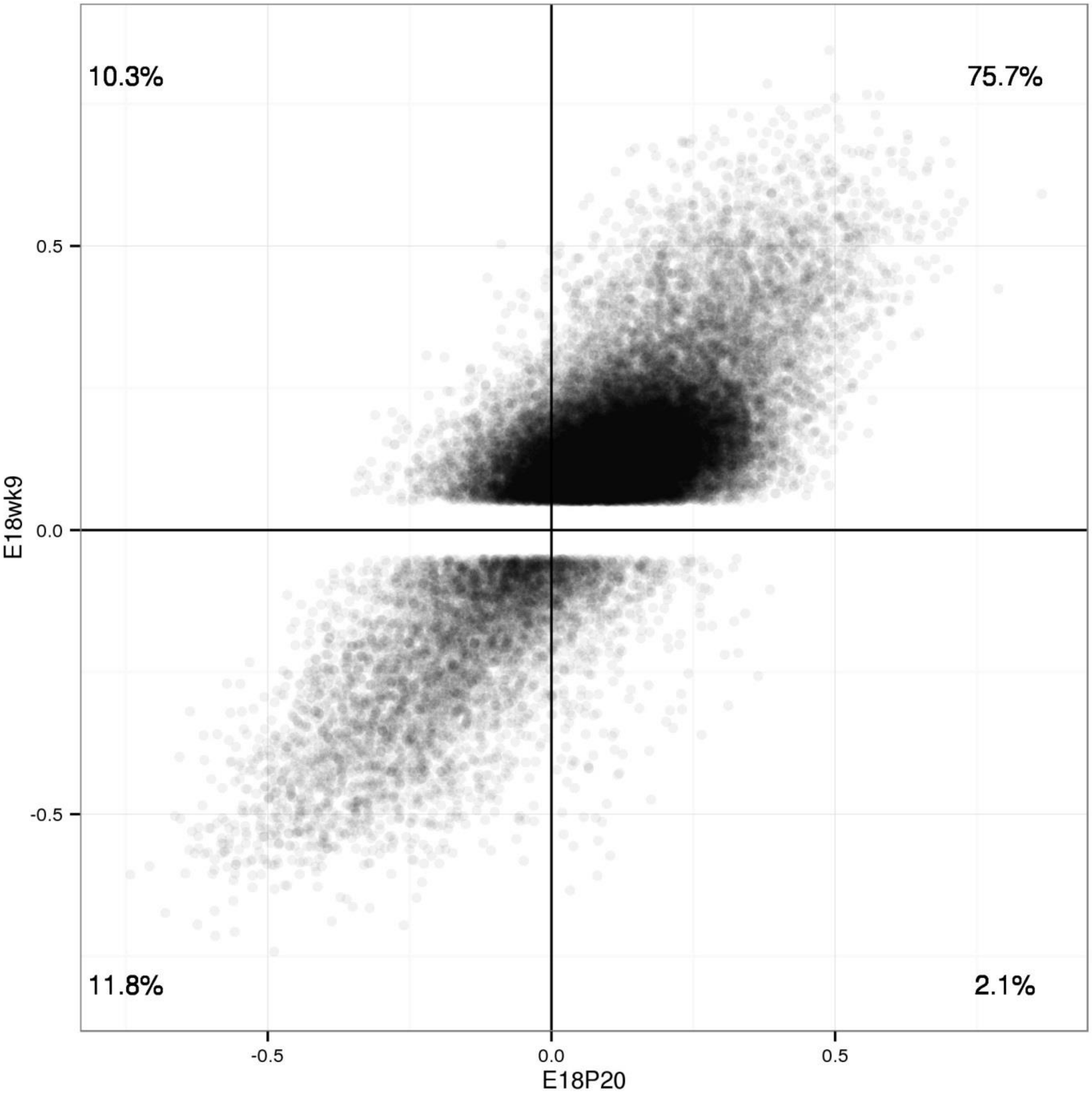

Figure S5: Comparison of discovery and time-course RRBS datasets

To determine reproducibility of the RRBS datasets, we plotted methylation values for each CpG for the discovery vs. the time-course datasets. The methylation values fall predominately along the 1:1 line with 87.5% of CpGs falling in the lower-left or upper-right quadrants.
