# Supplementary figures and images for: Extensive Epigenetic Changes Accompany Terminal Differentiation of Mouse Hepatocytes After Birth
Source: G3 (Bethesda). 2016 Sep 21;6(11):3701–9. doi: 10.1534/g3.116.034785 (PMC5100869; doi:10.1534/g3.116.034785)

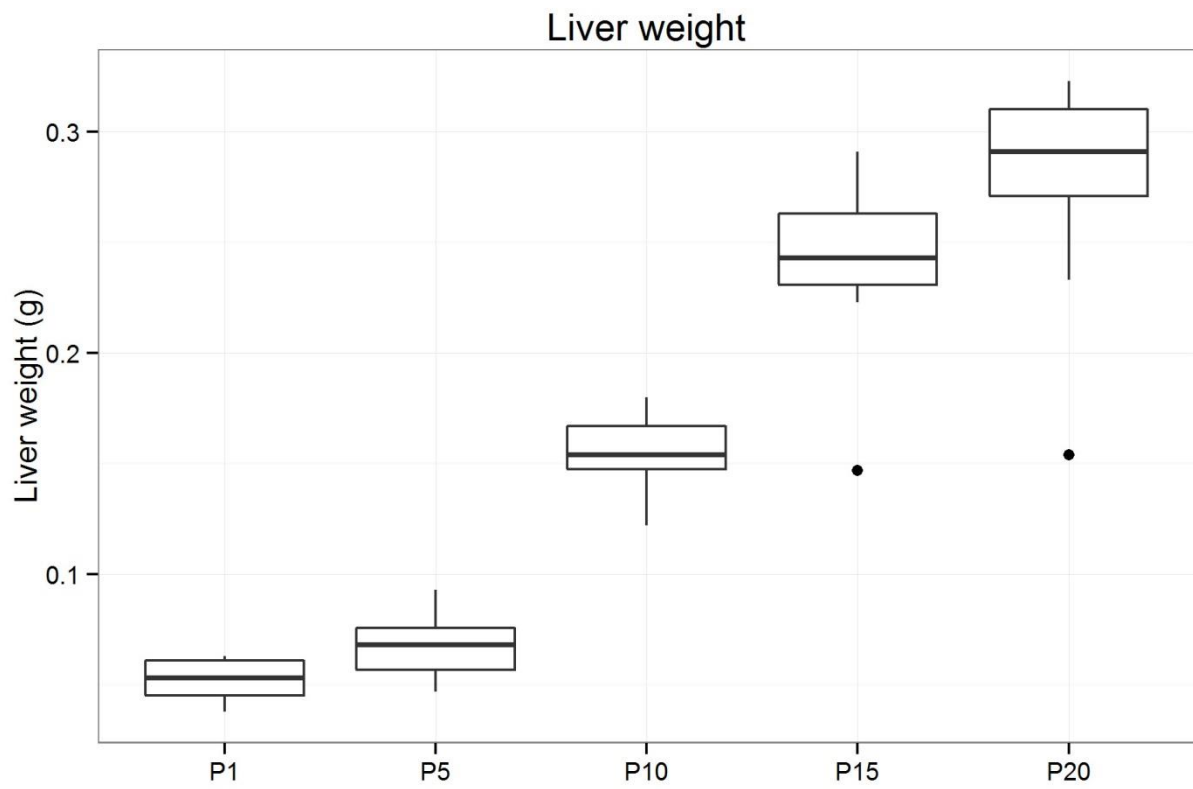

Figure S6: Liver weight from P1 through P20

The mean liver weight from P1 through P20 is presented.

Supplement: Supplemental Material [file supp_g3.116.034785_FigureS6.pdf]
