# Supplementary material for: Extensive Epigenetic Changes Accompany Terminal Differentiation of Mouse Hepatocytes After Birth
Source: G3 (Bethesda). 2016 Sep 21;6(11):3701–9. doi: 10.1534/g3.116.034785 (PMC5100869; doi:10.1534/g3.116.034785)
Supplement: Supplemental Material [file supp_g3.116.034785_FigureS7.pdf]

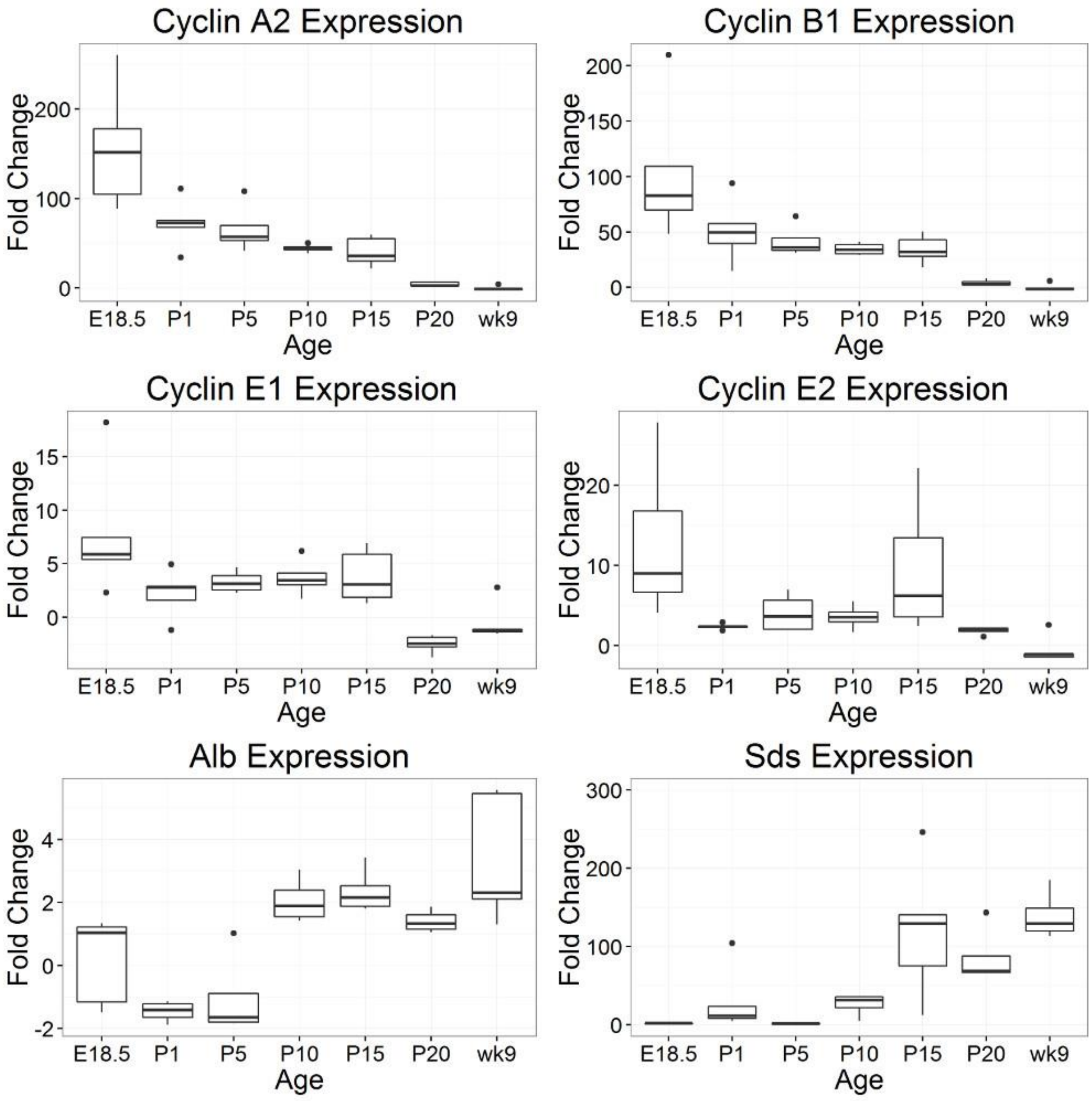

Figure S7: Gene expression of *Cyclins* and hepatocyte expressed genes

To estimate the rate of cellular division we quantified the expression of *Cyclin* genes. *Cyclin* expression was greatest at E18.5 and decreased with age. The expression at P20 was similar to the adult values. *Albumin* expression decreased from E18.5 through P5, then increased to roughly twice the E18.5 value. *Sds* expression (a marker of differentiated hepatocytes) begins to increase between P5 and P10 with a large increase between P10 and P15.
