# Supplementary material for: Extensive Epigenetic Changes Accompany Terminal Differentiation of Mouse Hepatocytes After Birth
Source: G3 (Bethesda). 2016 Sep 21;6(11):3701–9. doi: 10.1534/g3.116.034785 (PMC5100869; doi:10.1534/g3.116.034785)
Supplement: Supplemental Material [file supp_g3.116.034785_FigureS9.pdf]

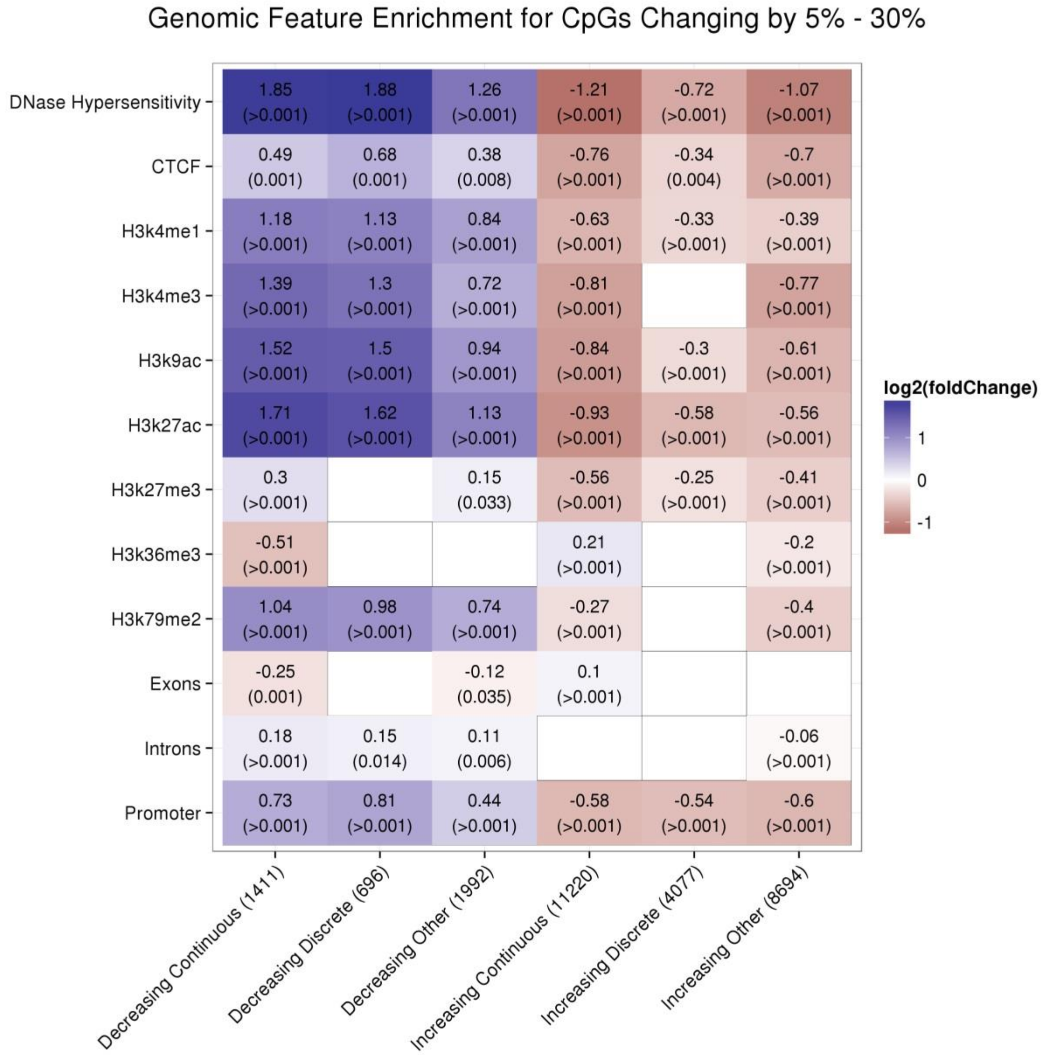

Figure S9: Enrichment for CpGs changing by between 5% and 30% in different genomic contexts.

We calculated enrichment values for genomic contexts for CpGs changing by between 5% and 30%. The CpGs were grouped by direction and pattern of change as described in the methods. Significant enrichment values are shown. We generated p-values, included beneath the fold change values in parenthesis, for each by resampling the data 1000 times, while correcting for local CpG density as detailed in the materials and methods.
